# Non-empirical methods for ethics research on digital technologies in medicine, health care and public health: a systematic journal review

**DOI:** 10.1007/s11019-024-10222-x

**Published:** 2024-08-09

**Authors:** Frank Ursin, Regina Müller, Florian Funer, Wenke Liedtke, David Renz, Svenja Wiertz, Robert Ranisch

**Affiliations:** 1 Institute for Ethics, History and Philosophy of Medicine, Hannover Medical School, Carl-Neuberg-Strasse 1, 30625 Hannover, Germany; 2 Institute of Philosophy, University of Bremen, Enrique-Schmidt-Straße 7, 28359 Bremen, Germany; 3 Institute for Ethics and History of Medicine, Eberhard Karls University, Gartenstrasse 47, 72074 Tübingen, Tübingen, Germany; 4 Faculty of Theology, University of Greifswald, Am Rubenowplatz 2-3, 17489 Greifswald, Germany; 5 Faculty of Protestant Theology, University of Bonn, Am Hofgarten 8, 53113 Bonn, Germany; 6 Department of Medical Ethics and the History of Medicine, University of Freiburg, Stefan-Meier-Str. 26, 79104 Freiburg, Germany; 7 Junior Professorship for Medical Ethics with a Focus on Digitization, Faculty of Health Sciences Brandenburg, University of Potsdam, Am Mühlenberg 9, 14476 Potsdam, Golm, Germany

## Abstract

Bioethics has developed approaches to address ethical issues in health care, similar to how technology ethics provides guidelines for ethical research on artificial intelligence, big data, and robotic applications. As these digital technologies are increasingly used in medicine, health care and public health, thus, it is plausible that the approaches of technology ethics have influenced bioethical research. Similar to the “empirical turn” in bioethics, which led to intense debates about appropriate moral theories, ethical frameworks and meta-ethics due to the increased use of empirical methodologies from social sciences, the proliferation of health-related subtypes of technology ethics might have a comparable impact on current bioethical research. This systematic journal review analyses the reporting of ethical frameworks and non-empirical methods in argument-based research articles on digital technologies in medicine, health care and public health that have been published in high-impact bioethics journals. We focus on articles reporting non-empirical research in original contributions. Our aim is to describe currently used methods for the ethical analysis of ethical issues regarding the application of digital technologies in medicine, health care and public health. We confine our analysis to non-empirical methods because empirical methods have been well-researched elsewhere. Finally, we discuss our findings against the background of established methods for health technology assessment, the lack of a typology for non-empirical methods as well as conceptual and methodical change in bioethics. Our descriptive results may serve as a starting point for reflecting on whether current ethical frameworks and non-empirical methods are appropriate to research ethical issues deriving from the application of digital technologies in medicine, health care and public health.

## Introduction

The methods and approaches to address ethical issues in medicine, health care and public health are challenged by the introduction of possibly disruptive digital technologies (Vayena et al. 2018). It seems plausible that the approaches of technology ethics may have influenced bioethics research in the same way as medical ethics has influenced digital ethics (Véliz 2019). Similar to the “empirical turn” in bioethics, which led to intense debates about appropriate moral theories, technology ethics might have an impact on current bioethics research. While empirical methods are well-researched in bioethics (Borry et al. 2006; Davies et al. 2015; Mertz et al. 2020; Wangmo and Provoost 2017), non-empirical methods and approaches are not. Concurrently, we are witnessing the “ethical proliferation” of, for example, artificial intelligence (AI) ethics, digital ethics, data ethics, internet ethics and robot ethics, which has recently been criticized by Sætra and Danaher (2022). They argue that we are already well-equipped with traditional ethical methods, theories and concepts; thus, we do not need to reinvent the wheel in the subdomains of technology ethics when applying its methodologies to bioethics. However, they have failed to highlight a toolbox of traditional ethical methodologies that is particularly suitable for the ethical analysis of digital technologies. This motivated our research to map non-empirical ethical methods and approaches that are presently used in bioethics research on digital technologies in medicine, health care and public health and are thus considered suitable by bioethicists. Our aim is to describe currently used methods for the analysis of ethical issues regarding the application of digital technologies in these fields. This article generates hypotheses for future studies while addressing the absence of a typology for non-empirical methods and exploring conceptual and methodological changes in bioethics.

We confine our work to non-empirical methods and related approaches, frameworks and theories, following an *ex negativo* approach in relation to empirical methods. Empirical methods from the social sciences have steadily gained ground in bioethics research (Davies et al. 2015) since the “birth of the empirical turn in bioethics” (Borry et al. 2005). This trend involves current endeavours to utilize computational methods for the analysis and exploration of bioethically significant phenomena in the digital era (Schneider et al. 2021). However, regarding bioethics’ normative dimension, not only empirical methods but also argumentative and conceptual methods are necessary to explore the digital space and its ethical implications for the health care domain (Salloch and Ursin 2022).

We discuss our findings within the context of ethical methods for the health technology assessment (HTA) and expect that this will provide us with methodological resources without “reinventing the wheel”. We differ from previous research by focusing primarily on articles reporting original non-empirical research and, to a lesser extent, on (systematic) reviews. Our descriptive results may serve as a starting point for reflecting on whether new ethical approaches and non-empirical methods are necessary to research ethical issues related to digital technologies in medicine, health care and public health, or whether we are well-equipped for future challenges of digital technologies with our traditional tools of thought.

## Materials and methods

We conducted a systematic journal review, in which we analysed the reporting of ethical approaches and non-empirical methods in research articles that have been published in high-impact bioethics journals. This study is focused on normative ethics with its analytical and action-guiding function in applied ethics (assessment and appraisal). Recognizing the absence of a universally accepted conceptual relationship between ethical methods, theories, frameworks, and approaches, we identified the need for a common understanding. All authors have agreed on working definitions. We understand moral theories as endeavours to guide understanding, explanation and reflection on moral decision-making (Flynn 2022). Unlike moral theories, ethical frameworks and approaches are tailored for specific domains and are often associated with practical guidance that support moral decision-making within those domains (applied ethics). Ethical frameworks or approaches are flexible variations of moral theories, allowing for adaptations and modifications, unlike comprehensive theories. They encompass values, norms, concepts and principles, sometimes with an analytical (assessment) and/or synthetic (action-guiding or appraisal) function.

An ethical method represents the cognitive procedures for analysing ethical issues or deriving ethically grounded normative decisions. While empirical methods are used in bioethics to collect data on aspects such as moral intuitions, attitudes or emotions, non-empirical methods in bioethics focus on the transition from theory to practice through deductive reasoning and serve an action-guiding function (Solomon 2004, pp. 820–822). Non-empirical methods might consist of first principles in moral theories together with factual descriptions of a particular morally problematic situation as part of a dialectical interplay between these principles and particular moral judgements. Typical examples of non-empirical methods in bioethics are inspired by coherentist epistemology, such as Rawls’ “method of reflective equilibrium” (Daniels 2020). Other examples include principlism with acceptable mid-level principles from various normative theories, such as in Beauchamp and Childress (2019), or casuistry as an analogy between paradigm cases and more problematic cases as illustrated in Jonsen and Toulmin (1988).

The aim of our systematic journal review was to map the ethical approaches, frameworks, theories and non-empirical methods that have been reported in research articles within bioethics journals. The process of identification, title and abstract screening and eligibility assessment is based on the systematic review methodology of Strech and Sofaer (2012). We did not pre-register our systematic journal review. The focus was on papers on digitalization in the last four complete volumes of ten high-impact journals starting from 2019. The data was subsequently charted in a predefined matrix, leading to an inductive clustering of similar non-empirical methods currently used in bioethics research.

### Selection of journals

To select key journals in the bioethics field, we included the ten journals with the highest impact factor in the category “Medical Ethics” from the Journal Citation Report of 2021 (Clarivate Analytics, *n* = 21 in total; see Table 1), speculating that new developments could be found here and given the absence of a distinct “Bioethics” category. Journals were then assigned randomly to each of the seven authors for screening.

### Inclusion and exclusion criteria for screening and eligibility assessment

Firstly, we included the last four complete volumes of the journals identified (2019–2022) up to February 17, 2023. This limitation was set to ensure a manageable scope of records, anticipating data saturation. We assumed that we would reach the point where adding more data would not significantly contribute to new insights or findings and aimed to prevent the analysis from becoming overly burdensome.

Secondly, we assessed all records formally according to the respective journal’s self-reported article types in order to include original research and exclude, e.g., opinion pieces. A list of article types for each journal included can be found in Table 1. If an author had difficulties in deciding whether an article should be included for the next step of full-text assessment, these cases were discussed among all co-authors. Difficult cases were articles with review methodologies and unique self-reported article types within some specific journal sections. The journal “BMC Medical Ethics”, for instance, publishes “reviews” as “research articles” (according to the authors’ judgement of the current work). The challenge concerning reviews is to decide whether they have an original component. For us, this meant developing an argument. Therefore, we included “review” articles only if they were aimed primarily at developing an argument based on the literature compiled, rather than descriptively gathering data within a literature review. Another rationale for constricting article types to original contributions is that “The American Journal of Bioethics” publishes a significant number of short commentaries, which are generally not self-standing original contributions disclosing methodological procedures and, therefore, were excluded from the substantive full-text assessment.

Thirdly, we substantively screened titles and abstracts of all remaining records, and included articles that dealt with the topic of digitalization in medicine, health care or public health. After intense discussions among all authors, we decided to apply a broad definition of digitalization including applications of AI, data science, clinical decision support systems, robots, electronic health records, mobile applications, telemedicine, and all procedures that apply computational methods within the health care domain, regardless of their current or (anticipated) future use. We excluded articles that were concerned with genomic data or financial aspects of research without a direct link to the practical application of digital technologies in medicine, health care and public health.

Fourthly, we considered articles eligible for full-text assessment if they dealt with ethical issues (Schofield et al. 2021) of any dimension of digitalization in medicine, health care or public health. During the full-text assessment, we excluded all articles that (a) did not report original research, such as commentaries, responses, editorials and debates (assuming that their argumentation represents an opinion piece), (b) did not address ethical issues in digitalization themes, (c) only descriptively generated empirical data by empirical methods as the objective of the research, (d) did not contain any non-empirical method, or (e) only reported literature reviews.

**Fig. 1:**
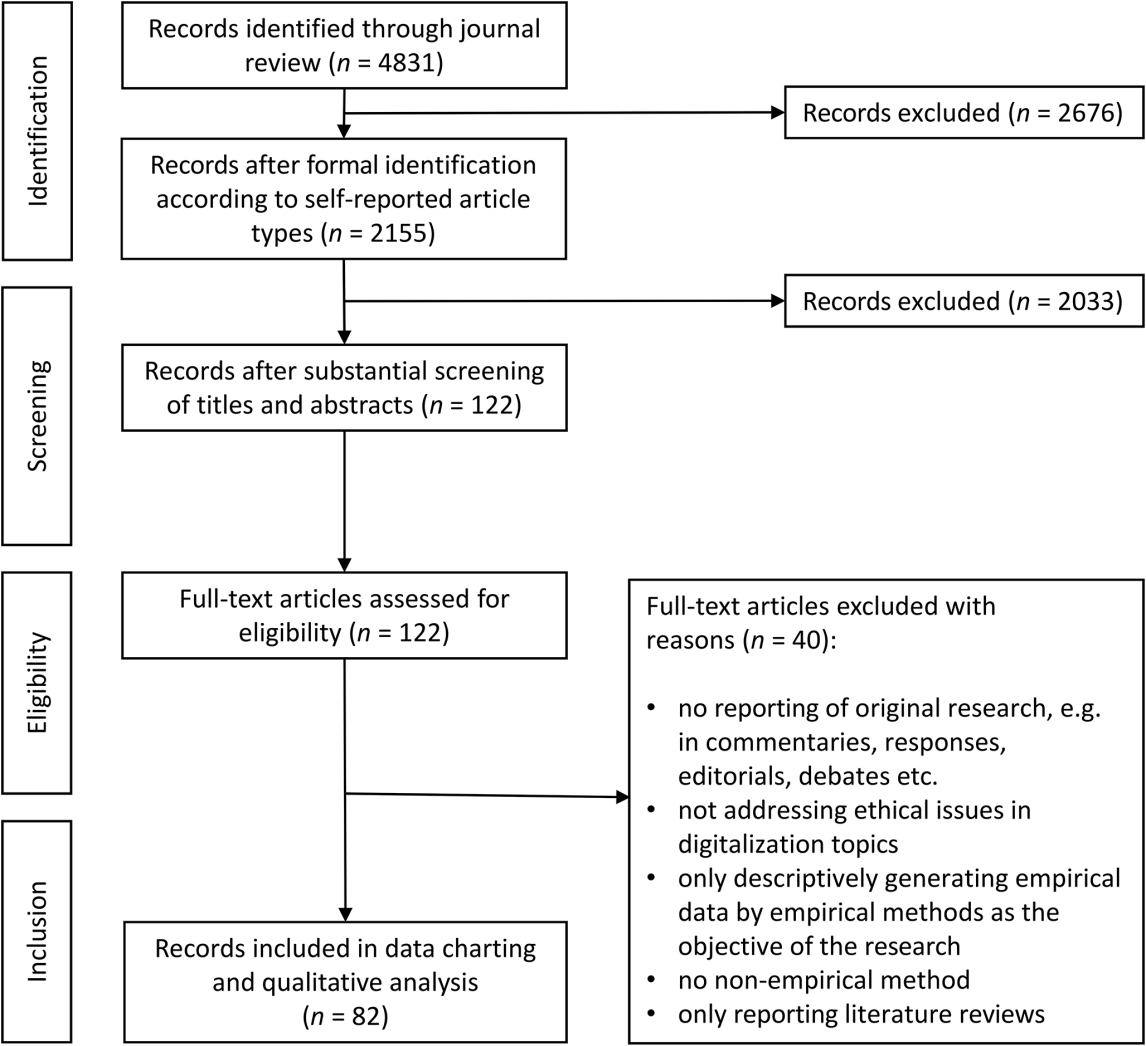
Flowchart according to the PRISMA statement

### Data extraction and charting

A draft data charting form was created and piloted by one author (FU) in order to refine it by discussion among all authors until consensus was reached. The selection of data items aimed at adequately representing the non-empirical methods applied in bioethics research on digitalization themes in medicine, health care and public health. The twelve final data items and respective types of extraction are as follows. The journal, title and type of the article as well as its year of publication were copy-pasted from the article PDF. Non-empirical methods and digitalization themes have been coded inductively from the abstract or main text (latent or in vivo). If there was no non-empirical method explicitly stated, then we have paraphrased it with verbal operators mirroring process coding with “-ing” words, e.g. “arguing”, “exploring” (cf. Miles et al. 2020, p. 66). The aim or research question, ethical approach, description of the non-empirical method, reference to non-empirical method in the literature, justification for choice of non-empirical method, limitations of non-empirical methods have been extracted from the abstract or main text as a citation. If there were additional empirical methods, then we extracted them from the abstract or main text as a paraphrase.

In terms of quality assurance, two authors (FU, RM) cross-checked and compared five randomly chosen datasets with the articles from which the data were taken. As a result of this review process, they harmonised the whole dataset. We have documented the procedure in detail, which can be accessed upon request from the corresponding author. Finally, we extracted the specific terms of non-empirical methods, frameworks, approaches and theories from the data charted and generated word lists.

## Results

The last four complete volumes of the top ten impact factor journals in the category ‘Medical Ethics’ (Clarivate Analytics 2021) contain *n* = 4,831 records in total. After applying the formal eligibility criteria, *n* = 2,155 records remained. After substantively screening titles and abstracts, *n* = 122 remained. We included *n* = 82 records in the data charting and qualitative analysis after full-text assessment of the remaining records. The journals “Accountability in Research-Policies and Quality Assurance” and “Developing World Bioethics” did not contain any article meeting our eligibility criteria. Most articles have been derived from “Bioethics” (*n* = 24) and “Journal of Medical Ethics” (*n* = 21). For a detailed account of the review process, see Tables 1 and Fig. 1.

**Table 1:** Journals included based on the Journal Citation Report (2021), article types and results of the screening procedure (2019–2022)

Journal	Article types and/or journal sections (self-reported) included	Identification: All records within the last four complete volumes (*n* = 4831)	Screening: Records after formal assessment of self-reported article types (*n* = 2155)	Eligibility: Records after substantive screening of titles and abstracts (*n* = 122)	Inclusion: Records included in data charting and qualitative analysis after full-text assessment for eligibility (*n* = 82)
The American Journal of Bioethics	target/research articles	1354	98	7	7
Journal of Law and the Biosciences	original articles	181	56	9	7
Journal of Medical Ethics	original research, feature articles, extended essays, current controversy	924	586	30	21
The Hastings Center Report	articles, essays (not: other voices, case reports)	479	187	12	7
Accountability in Research	articles, research articles	130	23	0	0
BMC Medical Ethics	research articles, research	539	401	23	7
Public Health Ethics	original articles, special symposium	126	104	5	4
Bioethics	original articles, special issue articles	546	423	25	24
Developing World Bioethics	original articles, articles	226	85	3	0
Journal of Bioethical Inquiry	original research, research, symposium: emerging technologies, symposium: COVID-19	326	192	8	5

### Non-empirical methods

Non-empirical methods are indicated with specific terms in *n* = 17 articles, with “ethical analysis” mentioned most frequently (*n* = 4). Similar methods are called “ethical assessment” or “ethical evaluation”. Other types of analyses include “risk-benefit analysis”, “descriptive analysis”, “conceptual-ethical analysis”, “multilayered conceptual analysis”, “philosophical-ethical reflection”, “theoretical trajectory” and “critical analysis of the academic debate and its shortcomings” (see Table 2). There are two “proof-of-concept studies” and “narrative syntheses” each. Only *n* = 4 articles referenced to *n* = 5 books or articles that describe the non-empirical method or methodology that has been applied in the respective article: the “critical interpretive synthesis” of Dixon-Woods et al. (2006) in D’Costa et al. (2020), the “ethical evaluation” of Whittlestone et al. (2019) in Rogers et al. (2021), and the “technological mediation approach” of Verbeek and Crease (2005) and Verbeek (2011) in Kudina (2019). Lupton (2020) referred to Frith (2012) in order to highlight the approach of “symbiotic empirical ethics” that informs theory and practice vice versa.

Non-empirical methods are combined with empirical methods in *n* = 23 articles. Empirical methods to gather data include phone and online interviews (Samuel et al. 2021); online surveys, focus groups and interviews (Lupton 2020); informal exploratory interviews and pluri-disciplinary discussions (Gaille et al. 2020); workshops (Winters et al. 2020); reviewing empirical data (Martens and Hildebrand 2021); scoping reviews (Jacquemard et al. 2020, 2021; Martani et al. 2020; Murphy et al. 2021); systematic review (Gesualdo et al. 2021); systematic app review (Sauerborn et al. 2021); and non-systematic literature reviews (Bhatia-Lin et al. 2019; Char et al. 2020; D’Costa et al. 2020; Gaille et al. 2020; Laacke et al. 2021; McCradden et al. 2022; Pyrrho et al. 2022).

Empirical methods to analyse data include structured qualitative content analysis (Sauerborn et al. 2021); inductive coding of ethical issues from case descriptions (Rogers et al. 2021); document analysis similar to systematic reviews of reasons (Ursin et al. 2022); and a comparison of an AI system’s output with answers obtained from “textbooks and from our ethicists” (Meier et al. 2022). The arguments were empirically informed in *n* = 4 articles (Martens and Hildebrand 2021; Rennie et al. 2020; Rossmaier 2022; Vandemeulebroucke et al. 2020).

The methodical procedures are described with the verbs “arguing”, “discussing” (ethically relevant issues, implications, concerns, research questions), “exploring” (ethical implications, moral/ethical arguments, limitations, notions of a concept, use of terminology), “concluding” (with recommendations, with reflections, by applying a concept, reasons), “examining”, “highlighting” (challenges, ethical issues, importance of a topic), “outlining”, “considering” (ethical issues, implications), “analysing”, “proposing”, and “suggesting”.

An explicit justification for selecting non-empirical methods is provided in *n* = 9 articles. The non-empirical methods with accompanying justifications encompass a synthesis of literature on trust justified by its unexplored nature (Samuel et al. 2021); ethical evaluation and AI use-case analysis justified by their relatively nascent status (Rogers et al. 2021); development of an applied ethics framework for electronic patient record development involving (hitherto disregarded) values (Jacquemard et al. 2021); exploration of moral arguments to map the current situation (Jacquemard et al. 2020); bottom-up mapping and discussion of ethical issues in response to tentative existing frameworks (Martani et al. 2020); multilayered conceptual analysis to dissect issues into distinct themes (Gaille et al. 2020); incorporation of social theory into bioethical assessments for “symbiotic empirical bioethics” through drawing on empirical findings (Lupton 2020; Vandemeulebroucke et al. 2020); and the use of a novel type of risk-benefit analysis due to the inadequacy of traditional human subject protections during a public health emergency (Martinez-Martin et al. 2020).

**Table 2:** Non-empirical methods for bioethics research on digital technologies in medicine, health care and public health

Non-empirical methods	• Conceptual-ethical analysis • Critical analysis of the academic debate and its shortcomings • Descriptive ethical analysis • Ethical analysis • Ethical assessment • Ethical evaluation • Multilayered conceptual analysis • Philosophical-ethical reflection • Risk-benefit analysis • Theoretical trajectory
References to external sources describing non-empirical methods	• “Critical interpretive synthesis” for systematic reviews: hermeneutics and argumentation analysis of Dixon-Woods et al. (2006) in D’Costa et al. (2020) • “Ethical evaluation” as a research roadmap that highlights ethical tensions between normative principles (“analytical lens”) of Whittlestone et al. (2019) in Rogers et al. (2021) • “Symbiotic empirical ethics” with overlaps but not similar to reflective equilibrium and pragmatic hermeneutics of Frith (2012) in Lupton (2020) • “Technological mediation approach” as a framework to analyse the roles and relations between humans and technology (“analytical lens”) of Verbeek (2011); Verbeek and Crease (2005) in Kudina (2019)
Justifications for selecting non-empirical methods	• Dissecting issues into distinct themes (Gaille et al. 2020) • Hitherto disregarded values (Jacquemard et al. 2021) • Inadequacy of traditional human subject protections (Martinez-Martin et al. 2020) • Incorporation of new theories into bioethical assessments (Lupton 2020; Vandemeulebroucke et al. 2020) • Nascent status of new use cases (Rogers et al. 2021) • Response to tentative existing frameworks (Martani et al. 2020) • To map the current situation (Jacquemard et al. 2020) • Unexplored nature of literature (Samuel et al. 2021)

### Ethical approaches, frameworks, concepts, and theories

There are references in *n* = 52 articles to ethical approaches, frameworks, concepts or theories (see Table 3). Two articles intentionally rejected frameworks because standard (principled) frameworks “are not necessarily appropriate in the context of a pandemic” (Martinez-Martin et al. 2020) and “design bioethics does not commit itself to a particular theoretical framework” (Pavarini et al. 2021). The most frequent reference to ethical approaches (*n* = 6) was made to the principlism of Beauchamp and Childress (Braun et al. 2021; Laacke et al. 2021; Meier et al. 2022; Parsons 2021; Richie 2022; Ursin et al. 2022). A combination of principles with other concepts has been addressed in a variety of publications: prioritarianism with three principles (Winters et al. 2020); trust (Kerasidou et al. 2022; Milne et al. 2021; Segers and Mertes 2022; Starke et al. 2021) and ecologies of trust (Samuel et al. 2021); safety, effectiveness, acceptability, and service-specific concerns derived from non-maleficence (Parsons 2021); privacy, confidentiality, autonomy, beneficence, non-maleficence, justice and respect for people (Aboujaoude 2019); autonomy, beneficence and justice (Porsdam Mann et al. 2021); autonomy, justice, humanity, lucidity and fidelity (Frittgen and Haltaufderheide 2022); fairness (Greely 2020; Grote 2022; Terrasse et al. 2019); justice (Hendl and Roxanne 2022); equality and inequality (Voigt 2022); benefits and harms/risks (Parker et al. 2020; Terrasse et al. 2019); liberty and privacy, responsibility, equity, fairness and justice (Parker et al. 2020); and explicability (Ursin et al. 2022).

We distinguish between general and specific ethical frameworks, in that specific ethical frameworks refer to an exact reference in the literature, while general ones do not. General ethical frameworks refer to the goals of medicine and green bioethics (Richie 2022); philosophical bioethics and human-computer interaction (Grote and Berens 2021); health care ethics (Rogers et al. 2021); care ethics and dehumanization (Palmer and Schwan 2021); ethics of contact tracing (Schaefer and Ballantyne 2022); moral responsibility (Grote and Berens 2020; Kempt and Nagel 2022; Miller and Smith 2021); standard accounts of expert decision-making and standards of traditional medical knowledge (London 2019); social epistemology and fairness (Grote and Berens 2020); forward-looking responsibility (Sand et al. 2021); paternalism (Grote and Berens 2020; Kühler 2021; McDougall 2019); empowerment (Segers and Mertes 2022); concepts of value-sensitive and -flexible design (McDougall 2019); the ideal of shared decision-making (Holm 2021; McDougall 2019); clinical equipoise (Grote 2022); informed consent (Grote 2022; Konicki et al. 2022); doctor-patient relationship (Konicki et al. 2022); decolonialism and Indigenous data sovereignty (Hendl and Roxanne 2022); participatory governance (Milne et al. 2021); capability approach and dignity-based arguments (Jecker 2021); benefits and harms (Parker et al. 2020); and more-than-human theories, such as new materialism, sociomaterialism and critical posthumanities (Lupton 2020).

Specific ethical frameworks reference Foucault’s notion of Bentham’s panopticon in terms of surveillance and self-regulation (Couch et al. 2020); Manson’s and O’Neill’s system of accountability in medicine (Felder 2021); Noggle’s concept of a functionally normal belief and Gendler’s concept of an alief (Martens and Hildebrand 2021); Walker’s expressive-collaborative model of morality, Tronto’s ethico-political analysis of care and Feenberg’s critical constructivism of technology (Vandemeulebroucke et al. 2020); Young’s and Fricker’s work on structural injustice including epistemic injustice as well as public health ethics (Sauerborn et al. 2021); Powers and Faden’s framework for structural injustice (Rossmaier 2022); Hinton’s concept of trust (Alvarado 2021); Feinberg’s public health approach (Raus et al. 2021); Emanuel et al.’s research ethics framework (Rennie et al. 2020); and a self-developed applied ethics framework (Jacquemard et al. 2021).

The codified ethical frameworks are influenced by law and health policy (Liddell et al. 2021); research ethics guidelines (Bhatia-Lin et al. 2019; McCradden et al. 2022); principled privacy protections (Bhatia-Lin et al. 2019); AI ethics frameworks (Rogers et al. 2021; Ursin et al. 2022); the Belmont Report (McCradden et al. 2022); the US Food and Drug Administration’s (FDA) policy for health and wellness apps (Kasperbauer and Wright 2019); and ethical and legal concepts of data ownership (Konicki et al. 2022).

**Table 3:** Ethical approaches in bioethics research on digitalization in medicine, health care and public health

Principled approaches	• Acceptability • Autonomy • Beneficence • Confidentiality • Dignity • Effectiveness • Equality • Equity • Explicability • Fairness • Fidelity • Humanity • Inequality • Justice • Liberty • Lucidity • Non-maleficence • Privacy • Responsibility • Safety • Trust
Theories	• Care ethics • Critical posthumanities • New materialism • Sociomaterialism • Virtue ethics
General ethical approaches	• Benefits and harms • Clinical equipoise • Decolonialism • Dehumanization • Doctor-patient relationship • Empowerment • Ethics of contact tracing • Goals of medicine • Green bioethics • Health care ethics • Human-computer interaction • Ideal of shared decision-making • Indigenous data sovereignty • Informed consent • Moral responsibility • Participatory governance • Paternalism • Philosophical bioethics • Social epistemology • Standard accounts of expert decision-making • Traditional medical knowledge • Value-flexible design • Value-sensitive design
Specific ethical approaches	• Emanuel et al.’s research ethics framework • Feenberg’s critical constructivism of technology • Feinberg’s public health approach • Foucault’s notion of Bentham’s panopticon in terms of surveillance and self-regulation • Hinton’s concept of trust • Manson and O’Neill’s system of accountability in medicine • Noggle’s concept of a functionally normal belief and Gendler’s concept of an alief • Powers and Faden’s framework for structural injustice • Self-developed applied ethics framework • Sen and Nussbaum’s capabilities approach • Tronto’s ethico-political analysis of care • Walker’s expressive-collaborative model of morality • Young and Fricker’s work on structural injustice including epistemic injustice and public health ethics
Codified ethical frameworks	• AI ethics frameworks • Ethical and legal concepts of data ownership • Law and health policy • Principled privacy protections • Research ethics guidelines • The Belmont Report • The US Food and Drug Administration’s policy for health and wellness apps

### Digitalization topics and themes

The most frequently mentioned topic was AI (*n* = 29), and includes sub-themes such as black-box algorithms, human-AI collaboration, death prediction, diagnostics, decision-making, the developing pipeline, disruptive innovations in health care, health and wellness apps, research ethics frameworks for AI, as well as patient autonomy and trust towards AI. The following themes appeared in the context of the COVID-19 pandemic: digital immunity passports, digital vaccination passports and, most commonly, contact tracing apps and wearables in the context of surveillance (*n* = 8). The data topic (*n* = 12) includes the sub-themes of health data research, data ownership, personal data, protection of privacy, electronic patient records and data trust models. The robots topic (*n* = 3) includes medical care robots, pets in dementia care and sex robots. Digital pills, mHealth, telemedicine and digital health each appeared three times. The following topics appeared only once: blockchain, computational pathology, virtual surgical planning, digital games for empirical research in bioethics, digital public commenting, digital tools for obtaining informed consent, digital twins, apps in general, health and wellness apps, facial phenotyping (in psychiatric genomics studies), disruptive innovations in health care, innovative technologies, a microfluidic chip for sperm sorting and its use for non-medical sex selection.

## Discussion

### The gap between non-empirical methods and approaches expected and found

Ethical methods are often conflated with frameworks and theories (McMillan 2018, p. 90). We speculate that this conflation arises because some approaches necessarily come along with their own methods (e.g. cost-benefit analysis and utilitarianism) and, therefore, an ethical approach (consisting of a method and a theory or framework) often incorporates both a heuristic (analytical) function for “ethical assessment” and a decisional (synthetic) function for “ethical appraisal”. Casuistry, for example, can be methodologically understood both as a heuristic tool and an alternative approach to moral theories (Düwell 2013, p. 55). We expected to find non-empirical ethical methods (that do not primarily rely on observation or data collection) in the screened literature, such as thought experiments, wide reflective equilibrium, logical analyses of arguments or concepts, as well as hermeneutical, dialectical and phenomenological approaches.

While it is suggested that we are encountering new ethical issues in the realm of health care digitalization (Vayena et al. 2018), our research revealed that the methods and approaches used in bioethics research on digital health care are comparable to those commonly found in general bioethics, as we could rarely see any new or de novo methods. However, our findings did not entirely match our expectations. We expected a greater plurality of non-empirical methods within bioethics research on digital technologies. Specifically, we anticipated the four principles of biomedical ethics with the occasional addition of explicability (Adams 2023; Beauchamp and Childress 2019; Floridi et al. 2018), in-depth conceptual analyses of ethical issues, casuistry (Jonsen and Toulmin 1988), HTA (Bellemare et al. 2018; Hofmann et al. 2015; Lysdahl et al. 2016; Patenaude et al. 2014), responsible research and innovation (RRI-Tools 2023), value sensitive design (Friedman et al. 2008), or the methodologies such as MEESTAR (a model for the ethical evaluation of socio-technological arrangements; Weber (2015).

These expected methods are not represented in the results, except for principlism and value sensitive design. Although we found “ethical assessments”, most of the ethical approaches of HTA that appear as promising candidates for ethical analyses and evaluations were absent in the articles included. These ethical approaches in HTA include (Bellemare et al. 2018; Lysdahl et al. 2016): principlism, casuistry, coherence analysis, wide reflective equilibrium, social shaping of technology, interactive HTA, the Triangular model, the HTA Core Model, and the Socratic approach (axiology). While some of these methodologies are more empirical than others, they differ predominantly in allowing for ethical assessment or appraisal or both. However, Bellemare et al. (2018) concluded that “compared with the scientific experimental paradigm, there are no settled proceedings for ethics in HTA nor consensus on the role of moral theory and ethical expertise hindering its integration”. Against the background of our findings, we conclude that the potential of HTA is not yet fully used in ethical analyses of digital technologies in medicine, health care or public health.

McCullough et al. (2004) distinguish six “basic appeals in argument-based ethics” of which we found with decreasing appearance: (1) ethical principles, above all, the principlism of Beauchamp and Childress (2019); (2) tradition and current practice standards (e.g. “goals of medicine”, “standard accounts of expert decision-making”, the “ideal of shared decision-making”, health data policies and AI ethics guidelines); (3) professional virtues (e.g. fairness, responsibility, fidelity). To a lesser extent we found (4) moral theories (virtue ethics, care ethics, ‘new materialism’, ‘sociomaterialism’ and ‘the critical posthumanities’ in Lupton (2020), but traditional moral theories, such as deontology or consequentialism, were not explicitly named); and (5) reflective equilibrium only once (Aboujaoude 2019). The approach of (6) casuistry was absent in our sample, although two “proof-of-concept studies” initially appeared to use this approach, they turned out to be reports on the development of specific digital applications for the health care domain (Meier et al. 2022; Pavarini et al. 2021).

We speculate that the gap between the non-empirical methods and approaches we expected and those we found can be explained by three hypotheses. Firstly, not just one specific method can serve the end of deriving ethically sound normative arguments. Interdisciplinary challenges, such as different academic backgrounds of bioethicists (Adler and Zlotnik Shaul 2012), biases in the publication and reporting of non-empirical research (Hofmann 2023), and a familiarity with methods of their original field, might lead bioethicists to gravitate towards these established methods. Secondly, the bioethics community seems to favour flexibility in their methods and approaches in light of their research objects (Adler and Zlotnik Shaul 2012). Not one specific method defines the field of study or ‘discipline’, as some would say, but the object of study. Therefore, different methods and approaches can serve bioethical research objectives. The widespread occurrence of flexible combinations of ethical principles seems to back this meta-scientific hypothesis. Thirdly, what counts as a method and which methods should be prioritized over others in the discourse are not as straightforward in bioethics as they are in the natural sciences.

### Lack of a typology of non-empirical methods in bioethics

In search of methodologies regarding how to proceed when conducting an “ethical analysis” of digital technologies in the health care domain, one might expect help in handbooks for bioethical methods (Arras et al. 2017; Ashcroft et al. 2007; Childress 1997, 2007; MacMillan 2018; Serna and Seoane 2016; Sugarman and Sulmasy 2010; Tomlinson 2012; Veatch and Guidry-Grimes 2020). What they have in common is that while they address specific bioethical issues paradigmatically, they rarely address issues of digitalization. So far, specific non-empirical methods to research this field are, thus, not to be found there. Reversely, specific ethical methods are also not in the scope of handbooks on ethical issues of, for example, AI as one branch of digital technologies in medicine, health care and public health (Dubber et al. 2020; Vallor 2022; Véliz 2021). Boddington (2023, p. 131) describes the common approach to “apply reasoning to test or correct one’s initial response” to a case of interest and highlights the issues arising from this simple model in terms of “selection and justification of any framework of ethical values and ethical theory used.” Rubeis (2024) uses the epistemic lenses of practice, relationship, and environment for his ethical appraisal of medical AI. In the domain of technology ethics, Nyholm (2023) relies heavily on wide reflective equilibrium, which the Stanford Encyclopedia of Philosophy counts as an appropriate method for practical ethics (Daniels 2020). In addition, Nyholm (2023) mentions the methods of “ethics by analogy”, “applying traditional ethical theories”, “ethics by committee”, and “empirical ethics”, while advocating for a mix of methods.

The lack of a typology of non-empirical methods in bioethics research is highlighted by the dearth of consistency in the nomenclature of ‘ethical analysis, assessment, evaluation, or appraisal’ and the relatively sparse references to concrete ethical methodologies in our sample. We only found “critical interpretive synthesis” for systematic reviews (Dixon-Woods et al. 2006) that might be conceived as hermeneutics and argumentation analysis, “ethical evaluation” (Whittlestone et al. 2019) as a research roadmap that highlights ethical tensions between normative principles (“analytical lens”), and “technological mediation approach” (Verbeek 2011; Verbeek and Crease 2005) as a framework to analyse the roles and relations between humans and technology (“analytical lens”). Thirty-two per cent of our sample included empirical methods in the research or the arguments were empirically informed, but only one article explicitly referred to Frith’s (2012) approach of “symbiotic empirical ethics” as a “practical methodology for integrating theory and practice”. She delineates her methodology against reflective equilibrium and pragmatic hermeneutics by following five steps: “setting out the circumstances (empirical part with data acquisition); specifying theories and principles; using moral theory as a tool of analysis; theory building; and, finally, making normative judgements”. However, Frith is also sparse in describing what “analysing” and “applying a theory to cases” means.

One might question whether there are any distinctive “ethical methods” at all beyond wide reflective equilibrium. If one browses through the pertinent handbooks on philosophical methodologies, one is usually left disappointed when searching for ethical methods (Cappelen et al. 2016; D’Oro and Overgaard 2017), because they mostly gather moral theories, ethical frameworks and approaches to be used as “analytical lenses”. It could be that moral theories necessarily come along with their specific methods “in the baggage”, and, therefore, no general typology of ethical methods (without the respective theories) is considered necessary. Vaughn (2020, p. 8) at least, provides a list with “methods of moral philosophy, which include, at a minimum, critical reasoning, logical argument, and conceptual analysis” (2020, 8).

Sidgwick (1907) once defined the “Methods of Ethics” as “obtaining reasoned convictions as to what ought to be done”, and distinguished the three moral theories: egoism, intuitionism and utilitarianism with their own distinct methods. MacMillan (2018, p. 182) took Sidgwick’s thinking further, defining bioethical methods as “techniques for reasoning about evidence, moral concepts, and combining them to build a case for a position” (2018, 182). His conception of moral reasoning is Socratic in terms of ‘drawing (conceptual) distinctions’ for normatively challenging practical ethical issues, necessarily resulting in an argumentative approach that relies on logical analyses.

Although principled, virtue-based, casuistic, narrative and feminist approaches as well as care ethics also provide “analytical lenses”, they do not necessarily prescribe a specific method on how to conduct the analysis or derive a decision (Veatch and Guidry-Grimes 2020, pp. 80–103). What makes matters even more obscure is that the same term (e.g. casuistry) is referred to as both a concept, an approach and a method (Veatch and Guidry-Grimes 2020, p. 102). Childress (2007, p. 16) is also not stringent in his conceptual usage of method, approach and framework when examining “[…] major types of principle-based methods (consequentialist, deontological, and pluralistic principlist methods), case-based methods, virtue ethics, ethics of care, and communitarian perspectives, along with some critical points from feminist perspectives and from rule-based theories” (2007, 16). Insofar as a general typology of non-empirical methods is needed, it may be found in “philosopher’s tool kits”. One clue might be the verbal descriptions of the methodological procedures in the articles we analysed, such as arguing, discussing, analysing and concluding.

Pfister (2017), for example, distinguishes between arguing (e.g. philosophical discussion, refutation by counterexamples, valid and invalid arguments, forms of arguments, fallacies), analysing (e.g. define terms, conceptual relations, concept analysis, explication), patterns of argumentation (e.g. *reductio ad absurdum*, infinite regress, transcendental argument, conclusion by analogy, thought experiment, abduction), logical analysis (e.g. category errors, analytic and synthetic propositions, *de re* and *de dicto*), and, most importantly, argumentation in ethics (e.g. decisions, norms and values, fallacies, patterns of argumentation, generalizability, means and ends, moral dilemmas, rights). These methods and conceptual background knowledge seem to belong to logical-philosophical propaedeutics, such as Daly (2010); Williamson (2020) or Baggini and Fosl (2010), more or less in the tradition of analytic philosophy. It is not surprising, therefore, that these methods emerge when focusing on argumentative normative bioethics.

### Conceptual and methodical change in bioethics?

We have already witnessed conceptual modifications to established ethical approaches due to technological innovations, such as the proliferation of explicability as a fifth principle of biomedical ethics (Adams 2023; Floridi and Cowls 2019) or an extended concept of health-related digital autonomy (Laacke et al. 2021) in the context of AI. Baker (2019) advocates the idea of “moral revolutions”, which can be triggered by techno-moral change (Danaher and Sætra 2023) due to morally disruptive technological innovations. Baker (2013) defines morally disruptive technological innovations as those which “undermine established moral norms or ethical codes” (Baker 2013, p. 59). While technological disruptions can entail moral disruptions, i.e. a change of acceptable moral stances, they might also bring forth new methods to anticipate and evaluate hitherto unknown ethical implications. Although forethought is desired, we acknowledge that there are epistemic hurdles, e.g. the Collingridge dilemma, i.e. the challenge of controlling technological developments, when their implications are still to be manifested, yet, once we know these implications, they are difficult to change (Kudina and Verbeek 2019).

We have also witnessed some methodological changes in bioethics research in recent years besides the ‘empirical turn’. Systematic review methodologies, for example, following the paradigm of evidence-based medicine (Goldenberg 2005; Kahrass et al. 2021; Mertz et al. 2017) aimed to circumvent biased normativity in being descriptive by mapping ethical issues (Schofield et al. 2021), reasons (Strech and Sofaer 2012) or arguments (McCullough et al. 2007). Whether or not the future lies in non-systematic and scoping reviews is a matter of ongoing debate (Birchley and Ives 2022; McDougall 2015; Parsons and Johal 2021). To conclude in relation to our topic, the import of methods from other disciplines to bioethics is possible but challenging. Besides conceptual and moral change, there might also be methodological change to keep pace with these evolving ethical landscapes, because the digitalization of medicine, health care and public health might include disruptive technological innovations.

Attention has recently been paid to at least disclose so-called bridge principles and, thereby, increase transparency and rigour on how normative claims are derived from empirical bioethics research (Kuehlmeyer et al. 2022). The reflection on the non-empirical methodological procedures and its thorough disclosure may further illuminate the intricate relationship between descriptive and prescriptive aspects of bioethics. Thus, a more robust integration of empirical insights and normative considerations is facilitated, because moral judgements “are always mixed judgments, based on both descriptive and prescriptive assumptions” (Düwell 2013, p. 27).

A distinction has to be made between the non-empirical methods applied in the research process and the reporting of the research in a scientific article. It became evident in the course of our analysis that some authors of argumentative and conceptual articles utilized non-empirical methods, yet, the presentation of these methods was rather vague. It is important to emphasize that the challenge may not reside solely in the absence of appropriate methodologies but rather in the deficiency of clear and comprehensive communication regarding the methods used. It is in the interest of both authors and readers of argumentative articles to enable readers to understand, assess and eventually replicate the methodological approaches taken.

Whether replicability, in addition to other methodological criteria for empirical and experimental science, should be an objective of non-empirical research in bioethics is disputable. These criteria may impose a methodological conception that may not be applicable to argumentative and conceptual research, for example, because they have different objectives. Without taking a stance on that issue, we acknowledge three positions: Firstly, one could argue that it is not sufficient to merely outline an argument; rather, it is equally imperative to explain how one arrived at that argument to permit replication in order to substantiate the validity of the argument. Secondly, on the contrary, developing arguments in moral philosophy does not allow for methodological rigor as in science due to its intrinsically creative and, therefore, opaque character, for example, assuming that the reporting of the dead ends of chains of thought do not contribute to the validity of the final argument. Thirdly, the various ways of thinking, along with moral theories and ethical approaches, all culminate in a wide reflective equilibrium that is adequately represented in argumentative articles.

We want to highlight examples within our findings and beyond to give an outlook of bioethics research on digital technologies in medicine, health care and public health. Promising new approaches combine bioethics, design ethics, and concepts of science and technology studies (Shaw and Donia 2021) and go further than “applying ethical theory”, “translating ethics for practice” and “identifying ethical harms”. This can be achieved by acknowledging the peculiarities of both bioethics and technology ethics with approaches such as understanding digital health systems as sociotechnical systems (Makarius et al. 2020). It can also be achieved through socio-historical contextualization, such as that in Vandemeulebroucke et al. (2020); acknowledging the ethical implications of AI and robot narratives in the light of the critical posthumanities (Cave et al. 2020; Coeckelbergh 2021), such as in Lupton (2020); or by applying the technological mediation approach (Verbeek 2011; Verbeek and Crease 2005), such as in Kudina (2019). Another promising approach could be to unleash the potential of the methodologies of ethics assessment and appraisal of HTA (Bellemare et al. 2018).

MacMillan (2018, p. 169) argues that “bioethics can also progress via the introduction of new concepts that are enable (sic! ) new or neglected issues to be identified” (2018, 169). Just as concepts such as nudging, exploitation or coercion were borrowed from other disciplines and, thus, became moral concepts, sustainability of sociotechnical environments (van Wynsberghe 2021) or the moral-epistemic dimension of explicability (Herzog 2022) were also discussed in relation to medical AI ethics. Therefore, methodical change in bioethics may not be found in non-empirical methods themselves (the corpus of these methods may be saturated) but in conceptual work for the new challenges of digitalization in medicine, health care and public health. The relatively new topics found, i.e. AI, health data, contact tracing and robots, seem to support the need for conceptual clarification before normative assessments are possible.

## Limitations

We focused on the reporting of non-empirical methods in original research within high-impact medical ethics journals to identify which ethical methods and approaches are held suitable to analyse, evaluate and assess the digitalization of the health care domain. We acknowledge several limitations. One that arose from our focus on the Clarivate Analytics category system is that there are journals missing that clearly have a focus on medical ethics, but are categorized differently (such as Cambridge Quarterly or Medicine, Health Care and Philosophy). However, we wanted a formal criterion for the field of medical ethics within bioethics. Original research on ethics in medicine, health care and public health is also occasionally published in clinical, informatics or philosophical journals.

In terms of generalizability, the question arises as to whether our results can be extended beyond the domain of digital technologies in medicine, health care and public health. Further investigation is needed in this regard to clarify the status of bioethics as a discipline or field of study that is determined by its methods or by its objects (Hofmann 2024). It remains to be seen whether bioethics and the phenomenon of digital technologies in medicine and health care possess unique characteristics that make them stand out within the ethics of technology.

## Conclusions

This study delved into the landscape of non-empirical methods, ethical approaches, frameworks, concepts and themes within the realm of health care digitalization, as reflected in recent publications from top-tier medical ethics journals. Our findings revealed that while non-empirical methods play a role in addressing the ethical complexities of digitalization, their utilization is diverse and often intertwined with empirical methods. Ethical analyses, evaluations and assessments emerged as key strategies employed by scholars to navigate the ethical issues of digital technologies in medicine, health care and public health. However, there is room for improved explanation regarding how exactly one should proceed when conducting an ethical analysis.

The breadth of ethical frameworks and concepts referenced in the articles analysed underscored the multifaceted nature of the challenges posed by digitalization in health care. Scholars drew upon a rich tapestry of ‘analytical lenses’ from established approaches, such as Beauchamp and Childress’s principlism, to emerging theories such as critical posthumanities. Moreover, the incorporation of legal and policy frameworks, AI ethics guidelines and research ethics standards attests to the interdisciplinary nature of bioethics.

AI emerged as a dominant topic, reflecting its impact on medicine, health care and public health eventually to be considered as a disruptive technological innovation with implications for techno-moral change. The ethical implications of AI encompassed a spectrum of themes, from algorithmic transparency, explicability and accountability to patient autonomy and trust. The COVID-19 pandemic further spotlighted contact tracing apps, digital immunity passports and wearables as tools for surveillance and public health management. Our mapping also unveiled various digital technologies that warrant ethical scrutiny, including electronic health records, telemedicine, mHealth and robotics.
